# National implementation of a trauma-informed intervention for intimate partner violence in the Department of Veterans Affairs: first year outcomes

**DOI:** 10.1186/s12913-018-3401-6

**Published:** 2018-07-24

**Authors:** Suzannah K. Creech, Justin K. Benzer, Tracie Ebalu, Christopher M. Murphy, Casey T. Taft

**Affiliations:** 1 VA VISN 17 Center of Excellence for Research on Returning War Veterans and the Central Texas Veterans Healthcare System, Waco, TX USA; 2 Dell Medical School of the University of Texas at Austin, Austin, TX USA; 3 National Center for PTSD, VA Boston Healthcare System, Boston, MA USA; 4 University of Maryland, Baltimore County, Baltimore, MD USA; 5 National Center for PTSD, VA Boston Healthcare System, Boston University School of Medicine, Boston, MA USA

**Keywords:** Veterans, Intimate partner violence, Aggression, PTSD, Trauma, Implementation

## Abstract

**Background:**

The U.S. Department of Veterans Affairs (VA) has recently implemented a comprehensive national program to help veterans who use or experience intimate partner violence (IPV). One important component of this plan is to implement *Strength at Home (SAH),* a 12-week cognitive-behavioral and trauma-informed group treatment designed to reduce and end IPV use among military and veteran populations.

**Method:**

The present study describes initial patient and clinician findings from the first year of a training program tasked with implementing *SAH* at 10 VA medical centers.

**Results:**

Results from 51 veterans who completed both pre- and post-treatment assessments indicate *SAH* was associated with significant pre- to post-treatment reductions in the proportion of veterans who reported using physical and psychological IPV toward a partner, the types of IPV used, and posttraumatic stress disorder symptoms. Overall, veterans reported high satisfaction with the quality and nature of services received, and with the program materials. In addition, 70% of sites and 34% of the 79 clinicians trained were successful in launching the program in the first year. The mean number of days between site training and initiation of the first group session was 135.86 (*SD* = 63.16, range 72–252).

**Conclusions:**

Results suggest that the training and implementation program was successful overall. However, average length of time between in-person training and initiation of group services was longer than desired and there were three sites that did not successfully implement the program within the first year, suggesting a need to reduce implementation barriers and enhance institutional support.

## Background

Over the past decade numerous research studies have indicated that high rates of intimate partner violence (IPV) among U.S. military veterans may convey risk for physical and mental health problems, as well as social, occupational, and legal difficulties [1, 2]. Women veterans are at high risk for experiencing IPV compared to their civilian counterparts [3], and male veterans with mental health disorders, particularly posttraumatic stress disorder (PTSD), evidence high rates of IPV use compared to both civilians and other veterans who do not have mental health disorders [4]. In response to this issue, in 2012 the Department of Veterans Affairs (VA) convened a Domestic Violence (DV)/IPV task force to develop recommendations for a national program. One year later, the task force finalized 14 recommendations to expand screening, prevention, and intervention for women and men veterans, as well as to introduce a VA employee assistance program for employees experiencing IPV [5]. The recommendations also included adopting non-stigmatizing language, specifically “IPV use” instead of IPV perpetration and “IPV experience” instead of IPV victimization. This program is relatively unique among healthcare systems because it places special emphasis on providing treatment to those who may be using violence or aggression in their relationships. Specific to veterans who have used IPV in their relationships, one task force recommendation in particular was that the VA implement a pilot of the *Strength at Home* program (*SAH*).

Prior to the implementation of the VA DV/IPV taskforce recommendations described above, few VA hospitals offered any programming for veterans using IPV beyond general anger management. This manuscript describes program outcomes from the first year of a pilot training program to introduce *SAH* at 10 VA hospitals. *SAH* is a 12-week cognitive-behavioral and trauma-informed group treatment to end IPV in military and veteran populations. Based on the social-information processing model of IPV in military populations [6], *SAH* has led to reductions in the use of physical and psychological aggression in pilot, effectiveness, and efficacy trials [7–9]. Importantly, until the trial of *SAH* [9], no prior intervention had demonstrated efficacy in reducing IPV in a military or veteran sample in a randomized clinical trial [10]. In fact, randomized clinical trials of IPV interventions have been rare, and results from civilian samples have shown these programs to have very small benefits in reducing IPV relative to legal system monitoring alone [11, 12]. Treatment programs that are most commonly available, such as those viewing IPV exclusively as a gender-based expression of power and control [13], depart substantially from the *SAH* model, in part because they do not account for the influence of trauma exposure, do not programmatically address core themes linking trauma to relationship problems and abuse, and often discount the importance of mental health symptoms commonly linked with IPV [6, 14]. Trauma and trauma-related consequences such as PTSD are consistently among the strongest risk factors for use of IPV in both civilian and military populations, and are therefore important considerations in the treatment and prevention of IPV [4]. As noted in a recent review paper authored by 17 leading IPV treatment experts, many existing interventions are not grounded in empirical research evidence or best practices [15].

In light of the above considerations, *SAH* was developed with the goal of establishing a model IPV treatment program for military and veteran populations. *SAH* consists of twelve 120-min group sessions organized into four treatment phases designed to address deficits in social information processing that increase risk for IPV: 1) psychoeducation on IPV and common reactions to trauma; 2) conflict management skills; 3) coping strategies and negative thought patterns; and 4) communication skills. All treatment components use military and veteran relevant language and examples. Practice assignments are completed at home. For example, veterans complete anger analysis worksheets designed to facilitate early recognition of anger signs and cognitive distortions, as well as the use of new skills and strategies learned in group. Group sessions start with a review of the practice assignment completed by each group member with feedback and discussion provided by the group members. This is followed by the introduction of new skills.

Prior to enrollment in *SAH*, all veterans complete an intake assessment, during which the assessing clinician prioritizes rapport building and uses motivational interviewing skills to enhance readiness for treatment [6]. During that intake, veterans complete measures of recent IPV, PTSD symptoms, and alcohol misuse. Due to high co-morbidity with both PTSD and IPV, alcohol misuse is included to help clinicians assess whether this factor may contribute to the veteran’s use of IPV or may complicate treatment efforts [16, 17]. Although *SAH* was not designed as a treatment for PTSD, the program addresses the impacts of trauma and core themes underlying trauma that may relate to IPV in relationships. Therefore measures of PTSD symptoms are also included.

A key component of *SAH* is partner outreach to ensure that any affected partners receive hotline numbers and information about other resources, safety planning, and the opportunity to complete a telephone assessment regarding their IPV experiences. Partner outreach is an important component of IPV treatment programs because partners may not have had the opportunity to reach out to available sources of aid and support, and they may not be aware of the resources available to them in the community. Research has suggested this direct outreach is an often overlooked opportunity to support those who have experienced IPV [18].

Using anonymous program evaluation data from veterans who completed the program at 10 VA hospitals, this manuscript describes patient outcomes from the first year of the VA pilot roll-out of *SAH.* We also present information on our initial approach to implementing this trauma-informed treatment for IPV. Specifically, we report on clinician success in the training program (number of clinicians facilitating groups within the first year of the program). We report on the number of veterans initially enrolled in the program and their demographic information. We also examine changes in the number of different types of IPV used, changes in the prevalence of physical and psychological IPV, and changes in both PTSD symptoms and alcohol misuse among those veterans who attended the majority of the program sessions (8 or more). We also examined veteran satisfaction with the treatment.

## Method

### SAH training program overview

#### Training sites and participating clinicians

As part of larger implementation efforts following the VA DV/IPV task force recommendations, six hospitals were designated by the VA DV/IPV task force as “rapid implementation sites” for the program. An additional three sites were added, for a total of nine in-person *SAH* trainings attended by personnel from 11 different hospitals in the first year of the roll-out. All sites participated voluntarily in the training program. The six rapid implementation sites were chosen by the VA DV/IPV taskforce because these sites had evidenced early commitment to the development of programming to address DV/IPV. Three additional training sites were added; two of these three sites were considered to have evidenced success in implementing other DV/IPV programming (though they were not designated as rapid implementation sites for the national program). Another training site was chosen due to its prior research experience with *SAH;* however, this site subsequently dropped out of the training program and is not included in analyses. In addition, clinicians from two other hospitals in close proximity to one of the designated pilot sites also completed *SAH* training. In total, 10 VA hospitals and 79 clinicians received *SAH* training and were enrolled in the pilot training program. All sites were required to have an IPV Coordinator tasked with overseeing the program and at least 4 clinicians who would volunteer to receive the training and commit to facilitating groups. All clinicians were required to be licensed mental health providers at the VA (i.e. social worker, psychologist, marriage and family therapist, licensed professional counselor). The training expectation was that at least one group would be initiated at each site within six weeks of the in-person training.

#### Training process and content

Prior to the training, IPV coordinators received a brief pre-training orientation call to answer any questions they had regarding the training, program expectations, and implementation tasks. Training was delivered in person over two days by the three *SAH* master trainers who were involved in developing *SAH* as well as in the conduct of the original *SAH* clinical efficacy trial [8, 9]. Each master trainer was a licensed clinical psychologist with years of experience working with individuals who use IPV, experience treating veterans and military families, and detailed knowledge of the *SAH* curriculum. The training consisted of one day focused on the theoretical underpinnings, program evaluation, and general therapeutic approach of *SAH*, with approximately 3 h of training in the use of motivational interviewing skills with veterans who may be using IPV. Attention was paid to ethics and mandated reporting statutes as well as to basic information on risk for IPV, dynamics of IPV in veterans, and clinical work with individuals using IPV. This was followed by a second day of detailed training on how to facilitate each of the 12 *SAH* sessions, augmented with role-plays of critical skills. Clinicians were provided with a treatment manual that described in detail the theoretical aspects of *SAH* and included all treatment materials. Also included in the manual was an assessment protocol detailing how to conduct the *SAH* intake interview and partner calls, clinician fidelity checklists, IPV fact sheets, *SAH* research publications, advice and materials for administering the protocol with LGBTQ and women veterans, and sample materials for publicizing the program and communicating with the justice system. Shortly after the in-person training, all clinicians were invited to an assessment and program evaluation training call.

After facilitating each *SAH* session, clinicians used a fidelity checklist to track their completion of all required elements for that session (using a yes/no checklist). All clinicians were required to complete weekly clinical consultation with the lead program developer while they led groups, and IPV Coordinators were required to attend monthly implementation consultation calls. In addition, the master trainers were available by phone and email for supplemental consultation as needed. To successfully complete the training program, clinicians were required to: 1) attend the full 2-day *SAH* training, 2) complete at least 2 *SAH* groups, 3) attend and participate in at least 75% of consultation calls, and 4) return program evaluation materials. After completing these requirements, clinicians could be cleared to facilitate *SAH* groups without concurrent clinical consultation.

#### Program evaluation procedures

Demographic information, session attendance, and scores on the program evaluation measures completed by program participants at the intake assessment and again at session 12 were summarized by site clinicians, and de-identified data were sent to the coordinating site. Summary information on recent IPV gathered from calls with veterans’ partners was likewise sent to the coordinating site. As the purpose was program evaluation only, no identifying information was collected by the evaluation team. To facilitate de-identification of the data, each participant was assigned a unique code by clinicians, and the same code connected veteran and partner data. The institutional review board at VA Boston reviewed the procedures and determined that the program evaluation did not meet the criteria for human research and thus was exempt from further IRB review.

### Program evaluation measures

#### IPV

Recent use of IPV was measured with an adapted version of the Centers for Disease Control’s 2010 National Intimate Partner and Sexual Violence Survey [19]. This 30-item measure assessed four types of IPV behaviors: 1) Psychological Aggression (e.g. “I acted very angry towards my partner in a way that seemed dangerous”), 2) Coercive Control (e.g. “I tried to keep my partner from seeing or talking to their family or friends”), 3) Reproductive Control (e.g. “I tried to get my partner pregnant when they did not want to get pregnant”), and 4) Physical Aggression (e.g. “I slapped my partner”). At two time-points (pre and post-treatment), veterans and their partners reported whether or not the veteran had engaged in each IPV behavior in the past 3 months on a dichotomous scale: 0 (*no*), 1 (*yes*). Following the recommendations of Straus and colleagues, prevalence scores were then calculated by summing the number of positive responses for each subtype of IPV, and converting the sum into a dichotomous variable representing the presence or absence of each type of IPV in the past three months [20]. Prevalence scores indicate whether one or more acts on the subscale occurred in the past three months. Therefore, a score of one indicates one or more types of IPV behaviors measured on each subscale occurred, whereas a score of zero indicates none of these behaviors occurred. In accordance with previous methodology, veteran-reported and partner-reported items were compared and if either person reported that a behavior occurred it was coded as present [21]. Summary scores representing the number of subtypes of IPV (i.e., psychological, coercive, reproductive, physical) were then computed (possible range = 0–4).

#### PTSD symptoms

PTSD symptoms were measured with the PTSD checklist for DSM 5 (PCL-5) [22]. The PCL-5 is a 20 item self-report measure of PTSD symptoms in the past month. Items are rated on a 5 point Likert scale (0 = not at all, 4 = extremely) and participants endorse symptoms based on “a very stressful experience.” Items are summed with higher scores reflecting greater symptomatology in the past month. A cut score of 33 is suggested as indicative of probable PTSD [23]. The measure evidences good reliability (Internal consistency = .96; test-retest = .84), discriminant and convergent validity [23].

#### Alcohol misuse

The 10-item Alcohol Use Disorders Identification Test (AUDIT) was used to assess problem drinking behavior over the past year [24]. The AUDIT is a screening questionnaire with three questions on the amount and frequency of drinking, three questions on alcohol dependence, and four questions on problems caused by alcohol; responses (on a 0 to 4 point scale) are summed, and higher scores reflect greater levels of alcohol misuse. A cut score of 8 is suggested as indicative of problem or hazardous drinking [25]. The AUDIT has a reported median reliability coefficient of .83, and adequate construct and criterion related validity [26].

#### Treatment satisfaction

Veterans also completed a 10-question post-treatment satisfaction measure, which was based on the 8-question Client Satisfaction Questionnaire (CSQ) [27, 28]. The adapted measure inquired into patient satisfaction with *SAH* and services received. Patients rated their satisfaction on a 4-point scale (from 0 to 3), and also responded to an open-ended question regarding the most and least helpful aspects of the program. Analyses of the open-ended responses were outside the scope of the current project and are not reported here. The CSQ-8 has been shown to evidence excellent internal consistency and to correlate positively with self-reported symptom change, as well as client and therapist reported improvement [28].

#### Implementation outcomes

Hospital adoption was the length of time in days between receipt of the in-person training and the start of the first group session for each site. Clinician adoption was the length of time in days between receipt of the in-person training and the start of the first group session for each clinician. We also report the percentages of clinicians who fully or partially completed the training program, as well as those who dropped out of the training program and their reasons for drop-out. For those sites who were not successful in implementing the program in the first year, we report the reasons why this may have occurred.

### Analyses

Paired samples *t*-tests were used to determine the significance of pre to post-treatment changes in measures of patients’ use of IPV, PTSD symptoms and alcohol misuse. Effect sizes for repeated measures designs (Hedge’s *g*_*rm*_) were also calculated [29]; an effect size of 0.2 is considered small, 0.5 is considered medium, and 0.8 is considered large [30]. The McNemar Chi Square test (without continuity correction) was computed to examine changes in the proportion of veterans using each subtype of IPV from pre-to-post treatment. All participants with data from both the pre and post-treatment assessment were included in these analyses. The IPV data, as described in further detail below, represent the report of veteran-to-partner IPV based on either the veteran’s self-report or the partner’s report (where possible). However, in order to mitigate against the influence of differential sensitivity for some veterans (as partner reported data was not available for everyone), all IPV analyses were repeated using veteran report only. The pattern of significant findings was identical; therefore, we report analyses only for the combined veteran-partner IPV data.

## Results

### Implementation outcomes

#### Hospital adoption

One year into the pilot rollout, seven of the ten sites had initiated at least one *SAH* group and the mean number of days between receipt of the training and the first group session was 135.86 (*SD* = 63.16, range 72–252). Of the sites that did not successfully begin a group in the first year of the rollout, reasons were: difficulty establishing institutional buy-in (*n* = 1), change in duties of the IPV Coordinator (*n* = 1), and lack of referrals (*n* = 1).

#### Clinician adoption

With regard to clinician outcomes for the hospitals that did successfully begin a group, 27 (34.18%) clinicians had facilitated at least one group and six (7.59%) clinicians had completed all requirements of the training program. The mean number of days between receipt of the training by a clinician and the first group session led by the clinician was 175.82 (*SD* = 83.81, range 72–378). Of the trained clinicians who had not facilitated any groups by the end of the first year, 21 (26.58%) indicated they still intended to lead a SAH group while 31 (39.24%) indicated they were no longer participating in the training program. Reasons for therapist drop-out included: competing responsibilities/increase in workload (*n* = 5), difficulty recruiting veterans (*n* = 2), lack of supervisor approval (*n* = 2), leaving VA employment or current position (*n* = 6), participated in training as program leader or veterans’ justice outreach specialist (*n* = 7), and unknown/no response (*n* = 9).

### Patients

There were 132 male veterans who completed a baseline assessment for the *SAH* program and 89 of these veterans were justice-involved (66%), meaning they had been charged with a crime related to IPV and/or were part of a deferred adjudication program or veteran’s treatment court. The average age of veterans who entered the program was 43.13 (*SD* = 13.77, range = 22–77). All other demographic and military service history information collected is presented in Table 1. At intake there were 102 (67.5%) veterans meeting or exceeding the PCL-5 cutoff for probable PTSD and 44 (29.1%) meeting the AUDIT criteria for hazardous alcohol use.

**Table 1:** Demographics and military history of study sample (*N* = 132)

	Number	Percent
Race/Ethnicity		
White	77	57.0
Black or African American	50	37.0
American Indian or Alaskan Native	1	.7
Hispanic	8	5.9
Other	7	5.2
Relationship Status		
Married	60	44.4
Separated or Divorced	44	38.5
Single	23	17.0
Education		
Less than High School	5	3.8
High School Graduate	33	25.0
Trade School/Some College	75	56.8
College Graduate	10	7.4
Some Graduate School	2	1.5
Graduate Degree	7	5.3
Military Service Era		
Vietnam	23	17.0
Persian Gulf	42	31.1
Operation Iraqi Freedom	50	37.0
Operation Enduring Freedom	40	29.6
Operation New Dawn	3	2.2
Other	20	14.8

*Note*: Patients could select more than 1 race/ethnicity category as well as military service era. Education missing for 3

### Partners

There were 59 partner calls completed at intake, and 14 partner calls completed at post-treatment. No demographic information was collected from partners, only collateral information on their experience of IPV.

### Attendance

Of the 132 veterans who completed intakes for the program, the mean number of sessions attended was 7.26 (*SD* = 4.42, range = 0–12). There were 53 veterans who attended 9 or more sessions and thus would be considered program “graduates”; there were 14 veterans who attended 0 sessions. Attendance data was missing for approximately 42 veterans. There were 51 veterans who completed a post-treatment assessment. Of the 51 veterans with completed post-treatment assessments, the mean number of sessions attended was 10.58 (*SD* = 1.14, range = 8–12).

### IPV outcomes

Initial results from 51 veterans who completed both pre- and post-treatment assessments (see Table 2) indicate the *SAH* treatment program was associated with a significant decrease in types of IPV used (combining across all 4 types of IPV) from baseline (the 3-months prior to treatment) to post-treatment *t* (50) = 3.39, *p* < .01, Hedges *g*_rm_ = .56 (medium).

**Table 2:** Pre and post-treatment IPV, PTSD symptoms and alcohol misuse for veterans with completed data at post-treatment

Variables	N	M	SD	Range
IPV (Pre-treatment)	51	1.51	1.32	0 − 4
IPV (Post-treatment)	51	.80	1.15	0 − 4
PTSD symptoms (Pre-treatment)	48	46.40	23.82	0 − 85
PTSD Symptoms (Post-treatment)	48	37.58	21.08	0 − 74
Alcohol Misuse (Pre-treatment)	50	4.74	6.64	0 − 35
Alcohol Misuse (Post-treatment)	50	3.62	4.73	0 − 16
Satisfaction (Post-treatment)	47	20.75	3.48	8 − 24

*Note:* IPV refers to the summed presence or absence of physical, psychological, coercive control and reproductive control behaviors, using the max score reported by the veteran or their partner; PTSD measured by PCL5; Alcohol misuse measured by AUDIT; Post-treatment satisfaction: higher scores indicate greater satisfaction with services received. Range is the observed range

There was also a significant change in the proportion of veterans with self or partner reported physical IPV use from baseline to the post-treatment phase (McNemar Chi Square = 7.12, *p* < .01). Specifically, of the 18 veterans with self or partner reported physical IPV use prior to treatment, 78% (*n* = 14) had no reported physical IPV use at the post-treatment assessment. Results also revealed a significant change in the proportion of veterans with self or partner reported psychological IPV use from baseline to the post-treatment phase (McNemar Chi Square = 9.78, *p* < .01). Of the 33 veterans with self or partner reported psychological IPV use prior to treatment, 58% (*n* = 19) had no reported psychological IPV use at the post-treatment assessment. There was also a significant change in the proportion of veterans with self or partner reported coercive control behaviors baseline to the post-treatment phase (McNemar Chi Square = 4.55, *p* < .05). Of the 25 veterans with self or partner reported use of coercive control behaviors prior to treatment, 64% (*n* = 16) had no reported use of coercive control at the post-treatment assessment. The McNemar test for change in proportions for those with self or partner reported use of reproductive control behaviors was not significant; however, there were only two veterans with self or partner reported use of reproductive control behaviors at baseline.

### PTSD and alcohol outcomes

Results also indicate a significant pre-post-treatment decrease in PTSD symptoms, *t* (49) = 3.29, *p* < .01, Hedge’s *g*_rm_ = .39 (medium). Mean scores on the PCL5 decreased from 46.40 (*SD* = 22.42) to 37.58 (*SD* = 21.08) from pre-post-treatment. The change in alcohol misuse was not significant, *p* = .20; the mean AUDIT score was 4.74 (*SD* = 6.64) at pre-treatment and 3.62 (*SD* = 8.20) at post-treatment. Findings for PTSD symptoms were not hypothesized, as *SAH* is not a PTSD treatment, although results suggest that the trauma-informed nature of the intervention may have an impact on PTSD.

### Treatment satisfaction

Post-treatment satisfaction data indicates that veterans were generally satisfied with the program (see Table 3), as evidenced by a mean score on the positive end of the scale. More specifically, 72.3% of veterans who completed the group reported they would “definitely” recommend the program to a friend, 68.1% reported the program helped them deal more effectively with their problems, and 58% indicated that they found the program materials “very helpful.”

**Table 3:** Means (and Standard Deviations) for post-treatment satisfaction measure

Question	M (SD)	Range
How would you rate the quality of service you have received?	2.81 (.40)	2–3
Did you get the kind of service you wanted?	2.64 (.49)	2–3
To what extent has *SAH* met your needs?	2.39 (.74)	0–3
If a friend were in a similar program, would you recommend *SAH* to them?	2.70 (.51)	1–3
How satisfied are you with the amount of help you have received?	2.60 (.74)	0–3
Have the services you received helped you to deal more effectively with your problems?	2.66 (.52)	1–3
In an overall, general sense, how satisfied are you with the services you have received?	2.64 (.53)	1–3
If you were to need help in the future for these problems, would you come back to *SAH*?	2.60 (.54)	1–3
How helpful were the materials the therapists gave you to read?	2.53 (.58)	1–3

*Note*. *n* = 47. Range is the observed range

## Discussion

This manuscript describes the format, structure, and initial therapist and patient outcomes of the first year of a pilot rollout of *SAH* at 10 VA hospitals. Readers should evaluate the potential causal effect of *SAH* on patient outcomes in the context of a naturalistic program evaluation of veterans who attended at least some *SAH* sessions and completed a post-treatment assessment. Significant reductions in the number of types of IPV used and changes in the proportion of the sample who used physical and psychological IPV toward their partners suggest the training model and initial implementation efforts were effective. Moreover, veterans were satisfied with the treatment they received. Reductions in PTSD symptoms from pre to post-treatment also suggest that the program may be helpful in reducing PTSD-associated distress and symptomatology. With regard to therapist outcomes, 70% of sites and 34% of clinicians were successful in launching the program. Combined with the reductions in IPV and PTSD symptoms observed in veterans, this suggests that the training program was successful overall.

The average length of time between receipt of the training and facilitating the first group was longer than desired and there were three sites that did not successfully implement the program within the first year. This suggests a need for improved attention to the implementation strategy, and in particular how to best ensure institutional support. Prior research has shown that an important facilitator of successful VA implementation is the alignment of resources to support delivery of the new treatment [31]. For example, starting a new program requires substantial infrastructure support such as identifying space to hold a group, identification of billing codes, and application for clinic status in the official medical record. In addition, the availability of the new program must be advertised to referring providers, who in turn, require information on the program and how to refer veterans to the program. If this work begins only after receiving the training, as was the case at some sites, the time to start the first group can be considerably delayed, underscoring the need to initiate implementation support and outreach efforts prior to the in-person training.

The number of clinicians successfully completing the training program was also lower than desired and there was a high rate of clinician dropout, primarily due to change in position and workload. Collectively this suggests the need for enhanced efforts to improve institutional and leadership commitment to offering the program by protecting clinician time to participate and improving leadership understanding of the amount of time required. This is a similar finding to those from the early stages of other VA rollouts of evidence-based treatments [32]. Specifically, in the early stages of the VA rollouts of *Cognitive Processing Therapy* and *Prolonged Exposure*, there were lower rates of training completion when clinician trainee requirements were less defined. An application process with leadership concurrence was introduced and this had a positive and significant impact on adoption of the therapy [32].

In response to these challenges and drawing from the literature describing successful implementation of other treatments within VA, the implementation strategy was improved for a second cohort of seven sites currently under way. First, we are attempting to enhance commitment from the local sites by requiring sites and clinicians to apply to receive the training. This provides an opt-in process that may exclude sites that do not have institutional support, and increases the likelihood that site leadership members understand the staff and resource requirements to successfully implement the program. Second, we now offer pre-training calls to sites and set requirements regarding activities that need to be completed prior to training. This requirement is intended to help them conduct the pre-implementation work needed to shorten the time between training and the first group session. Third, we specifically encourage sites to begin outreach efforts prior to training. Fourth, we now emphasize team-based implementation in which all clinicians assist with outreach and referrals.

The observational research design of program evaluation imposes important limitations on the interpretation of findings. The lack of a control group limits our ability to account for regression to the mean in IPV and PTSD symptoms. The naturalistic process of assigning patients to groups may increase effects by excluding patients who are less likely to benefit from the treatment, and non-randomized assignment precludes an intent-to-treat analysis. However, a previous randomized controlled trial [9] demonstrated that *SAH* was more effective than enhanced VA treatment-as-usual (ETAU) and the current results are consistent with that earlier research. Second, the outcome measures were limited in scope, most notably because we obtained only prevalence and not frequency counts for specific acts of IPV. However, the summary scores used in the current study are closely modeled after a widely used scoring method for measures of IPV, labeled “variety scoring,” which involves dichotomizing reports for each specific act of IPV and then summing the number (or variety) of different aggressive behaviors used [33]. Third, the amount of data available from partners, who are generally considered to provide the most accurate reports of IPV, was quite limited. Finally, all participant data for the program evaluation came from men. Likewise, only male participants were included in the pilot and efficacy studies of *SAH*. Further research is needed on program efficacy with women.

The observed effect size for pre- to post-treatment reduction in IPV in the current evaluation (*g*_*rm*_ = .56) is reasonable compared to a prior randomized clinical trial of *SAH.* The current effect size is larger than the effect size found in the prior research control condition (Hedge’s *g* = .36 and .26 for physical and psychological IPV, respectively), but smaller than the prior research treatment condition (Hedge’s *g* = 1.02 and .84 for physical and psychological IPV, respectively). Some gap in efficacy is to be expected in initial broad-scale implementation as a result of clinician expertise, experience with the population and intervention, and treatment integrity. However, methodological factors likely contributed here as well. Most notably, the selection criteria for the original controlled trial required all participants to have engaged in recent physical IPV, whereas the implementation sample included participants with less recent IPV or only psychological IPV, thus reducing the potential magnitude of observed pre- to post-treatment reduction in physical IPV. In addition, the controlled trial also used more precise measures of IPV frequency. Importantly, in the implementation sample, the prevalence of any physical IPV at post-treatment for those who reported physical IPV at baseline (22%) is essentially identical to rate reported for *SAH* in the controlled trial (23%), and considerably lower than the rate observed in the treatment-as-usual control (43%).

## Conclusion

Preliminary results from the implementation of *SAH* suggest that the intervention can be effectively delivered in real-world clinical practice at the VA. The observed reductions in IPV use in this implementation evaluation were consistent with the reductions observed in a highly controlled clinical efficacy trial [9]. Given data indicating other IPV treatments have minimal effects on repeat IPV above and beyond arrest and legal system monitoring, this is a very promising outcome [10–12]. However, delays between the completion of training and the start of groups, as well as clinician and site drop-out were limitations. Results identified four opportunities to strengthen the implementation strategy in order to increase the impact of the intervention. These strategies are being evaluated in a second cohort of seven sites.

## Abbreviations

DV: Domestic violence
ETAU: Enhanced VA treatment-as-usual
IPV: Intimate partner violence
PTSD: Posttraumatic stress disorder
SAH: Strength at home
VA: Department of Veterans Affairs
